# Single and selective transport of Zr(IV) ions with trioctyl amine dissolved in kerosene using a multidropped liquid membrane technique

**DOI:** 10.55730/1300-0527.3463

**Published:** 2022-06-02

**Authors:** Fatma TEZCAN, Ramazan DONAT

**Affiliations:** Department of Chemistry, Faculty of Science, Pamukkale University, Denizli, Turkey

**Keywords:** Zr(IV) ion, transport, extraction, MDLM system, TOA

## Abstract

It is very important to develop a process for selectively extracting Zr(IV) ions from the solution medium to produce zirconium metal used in industry and nuclear reactors. In this study, the parameters affecting the extraction of Zr(IV) ions were investigated using a multidropped liquid membrane (MDLM) system. Trioctyl amine (TOA) dissolved in kerosene was used as a carrier ligand in the extraction of Zr(IV) ions by the MDLM technique. The effect of carrier concentration and stripping solution concentration, pH, and the temperature in the donor phase on the transport of Zr(IV) were investigated. The optimum condition for the transportation of Zr(IV) was a 0.50 M H_2_SO_4_ solution as donor phase, 0.10 molL^−1^ TOA as a carrier, and 0.10 M Na_2_CO_3_ as acceptor phase. The transport percentage of Zr(IV) was increased up to >99% and the calculated activation energy which is 6.36 kcal mol^−1^ indicated that the process was diffusion controlled by Zr(IV) ions. The results showed that the MDLM system, which resembles a bulky membrane system in shape, is a promising technique for the extraction of Zr(IV) ions from an acidic solution. It is more practical and effective than bulk, supported, or inclusion, and emulsion liquid membrane techniques.

## 1. Introduction

Heavy metals are released into the environment through industrial waste. Since metals are nondegradable and waste loads in the environment constantly increase. Some of these metals are carcinogenic and pose serious threats to the environment. Today, attention is paid not only to the removal of pollutants from wastewater; but also to their recovery for reuse [1].

Zirconium and zirconium compounds have admitted increasing attention due to recent fields of application. They are operating in several areas, and 95% of zirconium exhausted is in the form of zircon, zirconia, and zirconium chemicals. The main application areas are 10% ceramics, 47% foundry sands, 22% refractories, and the rest is building chemical facilities, electronic equipment, and nuclear reactors. Zirconium is an element that has a very small neutron absorption area and is used as an alloying material for uranium-zirconium (U-Zr), the main construction material for uranium cladding, with full nuclear requirements. Besides that, Zr metal is also resistant to high temperatures, and has corrosion resistance, good heat conductor, and strong mechanical properties. Zirconium chemicals have been taking a private interest as high-tech materials for industrial and scientific implementation on account of their mechanical, thermal, electrical, and chemical activity [2].

The separation and extraction of metal ions in a solution are one of the most common problems in the chemical industry. Efficient separation processes are required to obtain the best products for industrial scales and to both remove and recover toxic or valuable components from industrial wastes. Today, many separations techniques such as distillation, precipitation, crystallization, extraction, adsorption, and ion exchange are used for these processes.

The general principles of chemical precipitation in industrial wastes are based on the low solubility of heavy metal hydroxides, salts, or sulfur. However, the low solubility method is known as the less efficient method [3]. On the contrary, in the case of low concentration heavy metal waste processing methods, this is more effective.

Membranes and membrane processing techniques were first used for analytical applications in chemistry and biomedical laboratories. Since it has significant technical and commercial impacts, it has been developed very quickly as a field of conversion into industrial products and application to these methods [4,5].

Membrane processes have several advantages, namely, the separation and purification technique takes place at room temperature, can be carried out continuously, varies in nature, can be adjusted as needed, the resulting membrane can be reused, and is environmentally friendly because it does not have a bad impact on the environment.

When compared to other separation techniques, the liquid membrane (LM) technique shows better selectivity and efficiency by the asset of a movable and selectively representative agent [6]. A suitable choice of extractant determines the success of the extraction process [7]. In the LM technique, molecules or ions are initially extracted in the external phase and pass from the membrane phase to the inner phase with the help of the carrier molecule dissolved in the membrane phase. In the extraction process, complex compounds are formed between metal cations and solvents, which can be applied to zirconium in solution.

In this study, a multidropped liquid membrane (MDLM) system which is similar to the bulk liquid membrane system is used. Easy structure, absence of moving pieces, lightweight and transportability, flexibility in the process, high membrane capability, and high separation factor than mixer-settler are the benefits of this technique. This method reduces the operating time, the risk of contamination amounts of reagents, and the generated waste. This process can be applied to heavy metal contamination wastewater by the magnification of the donor phase and acceptor phase reactors [8].

The extraction of zirconium from a solution containing sulfuric acid using trioctyl amine (TOA), which is one of the tertiary amines, in the treatment of kerosene as a diluent was investigated by the MDLM system. There are very few reports of using amine extractors in the extraction of tetravalent Zr(IV) alone or as synergists for the extraction of Zr(IV) from the sulfuric acid solution using high molecular weight amines. These studies were carried out with different membrane systems. The MDLM technique is more modular, economical, and easier to calculate the kinetic data of the extraction of metal ions compared to other membrane systems. The carrier effect of TOA in the sulfated solution of the donor phase and the selective extraction of Zr(IV) in the presence of other metal ions were investigated by the MDLM technique. This story represents experimental data on the extraction of Zr(IV) from sulfate solution using TOA as an extractant in kerosene. Various extraction effects on Zr(IV) give further insight into the influence of various factors. The results of the kinetics of Zr(IV) ions transportation, thanks to MDLM containing TOA as a carrier and flowing over aqueous phases, are presented in this paper.

## 2. Materials and methods

### 2.1. Reagents and chemicals

Trioctyl amine (TOA) is used as the extractant that is purchased from Merck. The chemical formula of the TOA is C_24_H_51_N and the molecular weight is 353.67 gmol^−1^. Kerosene was used as a diluent that is purchased from TUPRAS, Turkey. ZrOCl_2_.8H_2_O, H_2_SO_4_, Na_2_CO_3_, TOA, and Arsenazo III were used straight as admitted that were purchased from Merck (Germany).

### 2.2. Experimental procedure

It is a transport mode facilitated by the extraction of metallic cations by liquid membrane techniques. There are three phases in these systems: donor, acceptor, and membrane. The membrane phase contains a carrier ligand that extracts metallic cations in the organic solvent. The organic phase, which passes through the donor phase (feed), complexes the extracting ligand carrier metallic cation and thus carries it to the organic phase, and the stripping of the metallic cation or regeneration is provided from the organic phase to the acceptor phase where decomplexing takes place. The processes are continuous, and the transport of metal cations extracted from the donor phase to the carrier acceptor phase is ensured.

**Figure 1:**
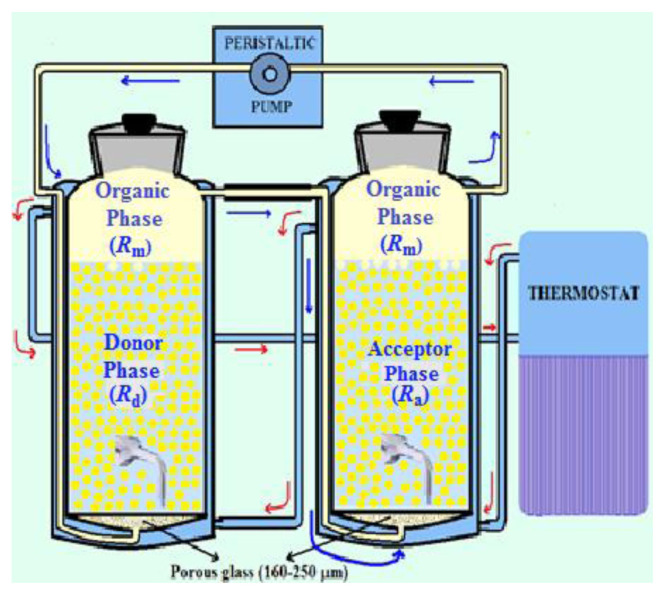
Schema of the measuring set with flowing MDLM system.

The experimental setup of the system is in Figure 1. It is made with porous glass in mind at the bottom of both reactors to eliminate mechanical agitation (in emulsion and bulk liquid membrane systems) and to disperse droplets (bubbles) more homogeneously.

In our MDLM system, the use of porous glass in the bottom of both reactors is in mind to eliminate mechanical agitation (in emulsion and bulk membrane systems) and to allow the organic phase to pass through the donor and acceptor reactors as droplets (bubbles). The carrier ligand TOA dissolved in kerosene has a lower density than that of the donor, so the acceptor and donor phases are located in the upper parts of the reactors. Donor and acceptor phases with carrier phases are immiscible. Zirconium ions are first located in the reactor on the left which is called as “Donor” phase, and they are bound to the carrier at the interface of both phases. Afterward, they are transported to the reactor on the right and stripped by the aqueous solution on the other reactor which is called as “Acceptor” phase. The main point of the MDLM system is the immobile donor-acceptor phase and mobile organic phase. In order to operate at a constant temperature, the cryostat device provides the circulation of tap water through the outer jacket of the entire reactor system.

### 2.3. Analytical instruments

Perkin Elmer brand Lambda 25 model spectrophotometer was used to determine the concentration of Zr(IV) ions in the donor and acceptor phases colorimetric. BT30-2J brand peristaltic pump to ensure the temperature of the system, flow, and pressure of the organic phase from the donor to acceptor phases with the Circu-WCR-P8 model cryostat device and WTW brand Microprocessor pH meter was used to determine the pH values.

### 2.4. Extraction experiments and theoretical model

The extraction (consecutively reaction) model is given in Eq (1).


(1) $$\text{Donor phase }(\text{A})\overset{k_{1}}{\to }\text{Org. phase }(\text{B})\overset{k_{2}}{\to }\text{Acceptor phase }(\text{C})$$

The concentration of Zr(IV) ions in A donor, B organic, and C in the acceptor phase, *k*_1_ and *k*_2_ are the reaction rate constants. In all parameters studied, the ln (*C*_o_/*C*_e_) change graphs were given against time for the concentration of Zr(IV) ions that passed from the donor phase to the organic phase over time, and the reaction rate constant (*k*_1_) values were calculated using Eq. (2) given below.


(2) $$\text{ln }\left(\frac{{\text{C}}_{0}}{{\text{C}}_{\text{e}}}\right)=k\text{t}$$

*C*_o_ and *C*_e_ refer to the concentration of Zr(IV) ions at the beginning as well at any time, respectively. The decreasing and increasing concentrations of Zr(IV) ions in all of the phases were plotted over time, and the *k*_2_, and were calculated with the help of Eqs. (3)–(5) given below [9].


(3) $$k_{2}=\frac{-\text{ln}\left(\frac{{\text{C}}_{\text{B}}^{\text{max}}}{{\text{C}}_{0}}\right)}{{\text{t}}_{\text{B}}^{\text{max}}}$$

(4) $$t_{\text{B}}^{\text{max}}=\frac{\text{ln}\left(\frac{{\text{k}}_{1}}{{\text{k}}_{2}}\right)}{{\text{k}}_{1}-{\text{k}}_{2}}$$

(5) $$C_{B}^{max}=C_{\text{o}}\;{\left(\frac{{\text{k}}_{1}}{{\text{k}}_{2}}\right)}^{\frac{{\text{k}}_{2}}{{\text{k}}_{1}-{\text{k}}_{2}}}$$

In Eqs. (3)–(5) $t_{B}^{max}$ and $C_{B}^{max}$ are the time and maximum concentration of Zr(IV) ions, respectively, to reach the maximum concentration in the organic phase.

For each experiment, depending on the reaction rate constants *k_1_* and *k_2_*, membrane inlet ( $J_{d}^{max}$) and membrane exit ( $J_{d}^{max}$) velocities respectively, the following Eqs. (6)–(9) are used [10].


(6) $${\left[\frac{{\text{dR}}_{\text{d}}}{\text{dt}}\right]}_{\text{max}}=-k_{1}\;{\left(\frac{k_{1}}{k_{2}}\right)}^{\frac{k_{2}}{k_{1}-k_{2}}}=J_{d}^{max}$$

(7) $${\left[\frac{dR_{m}}{dt}\right]}_{max}=0$$

(8) $${\left[\frac{dR_{a}}{dt}\right]}_{max}=-k_{2}\;{\left(\frac{k_{1}}{k_{2}}\right)}^{\frac{k_{2}}{k_{1}-k_{2}}}=J_{d}^{max}$$

(9) $${\left[\frac{dR_{d}}{dt}\right]}_{max}=+{\left[\frac{dR_{a}}{dt}\right]}_{max}\Rightarrow -J_{d}^{max}=J_{a}^{max}$$

Using Eqs. (10) and (11) given below, activation energy and extraction efficiency percentages of Zr(IV) ions were calculated according to the data obtained from experimental studies.


(10) $$ln(J)=ln(A)-\frac{E_{a}}{R}\left(\frac{1}{T}\right)$$

(11) $$Ext.\%=\frac{{\left[Zr(IV)\right]}_{acceptor}}{{\left[Zr(IV)\right]}_{donor}}\times 100$$

## 3. Results and discussion

### 3.1. Effect of TOA concentration on the extraction of Zr(IV) ions

The most commonly used amine extractors in extraction studies include amines and salts (primary amines, secondary amines, tertiary amines, quaternary ammonium salts). One of these extractors, TOA is very selective in various separation processes and easily dissolves in most organic diluents over a wide temperature range. The amine group-containing extractants used in extraction studies of Zr(IV) ions are listed in Table 1.

Metal precipitation can be defined as a process where metal ions react with other compounds to form a product of low solubility. Metal hydroxide precipitation is the most common example [15]. In the absence of ${\text{SO}}_{4}^{2-}$, Zr^4+^ hydrolysis can be initiated even in high acidity. Considering complexation in an aqueous solution, the pH ranges for precipitation will increase. The complex reaction with inhibits the hydrolysis of Zr^4+^ when pH increases, because the use of ${\text{SO}}_{4}^{2-}$ creates competition between ${\text{SO}}_{4}^{2-}$ and OH^−^ ions to react with Zr^4+^.

The predominant Zr forms in acidic sulfate solution with pH < 0.0 are Zr^4+^, Zr(SO_4_)^2+^, and Zr(SO_4_)_2_. However, the pH range for hydrolysis of active Zr forms in acidic sulfate solution with pH > 0.0 has increased and their chemical formula is $\text{Zr}{\left({\text{SO}}_{4}\right)}_{3}^{2-}$. Such situations are consistent with those reported in the literature. Selective recovery of Zr(IV) from the sulfuric acid leach solution by neutralization is not possible due to coprecipitation of other metals [16].

The extraction of Zr(IV) from H_2_SO_4_ solution using TOA was investigated in organic diluents such as kerosene. Kerosene was used as the diluent for the study due to its low toxicity, low cost, low density, and easy availability.

**Figure 2:**
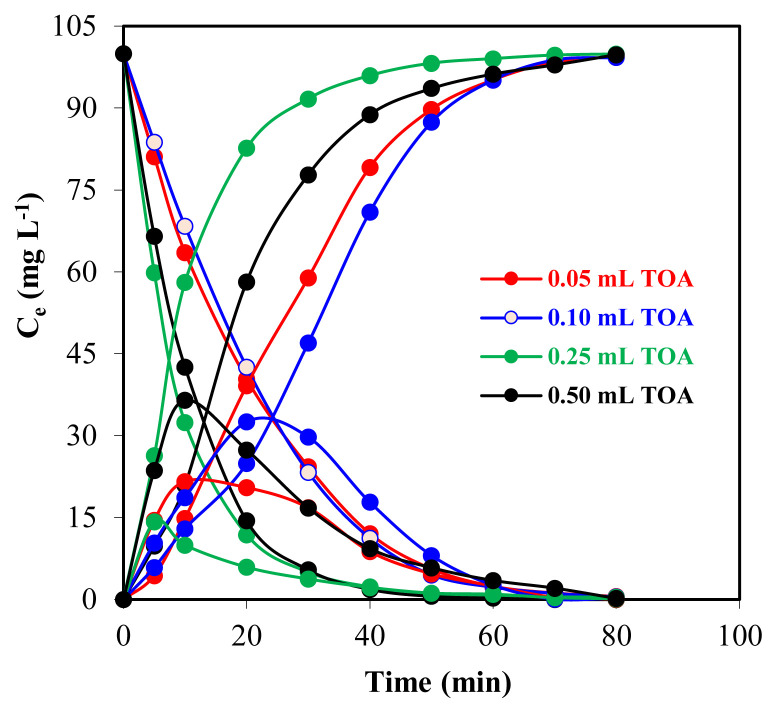
Graph of the change over time of the concentration of Zr(IV) ions in three phases with four different TOA concentrations in continuous extraction studies.

To investigate the effect of different TOA concentrations on the transportation of Zr(IV) ions, optimum conditions are given as follows: the volume of the donor, the acceptor, and the organic phase is 100 mL, initial Zr(IV) ion concentration of donor phase is 100 mg L^−1^ in 0.50 M H_2_SO_4_ solution, Na_2_CO_3_ concentration is 0.10 M in acceptor phase, TOA concentrations of organic phase dissolved in kerosene (0.05, 0.10, 0.25, and 0.50 mL for each experiment) are studied by setting the ambient temperature to 298.15 K, and the solution transfer rate of the peristaltic pump to be 25 mL min^−1^ (Figure 2).

**Figure 3:**
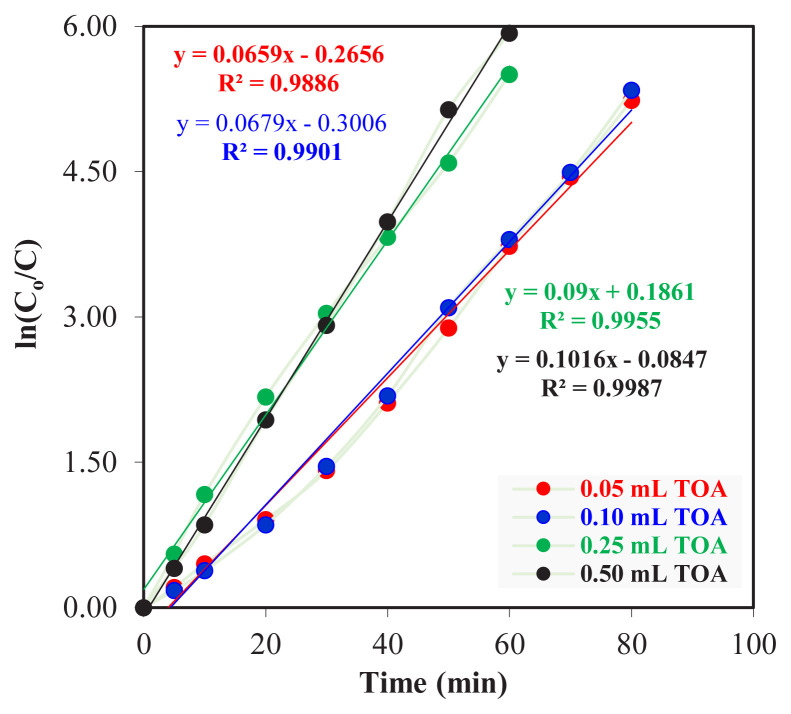
The graph of extraction kinetics of Zr(IV) ions for experiments carried out at different TOA concentrations.

In Figure 2, the effect of 0.050, 0.10, 0.25, and 0.50 mL TOA in kerosene on the change of Zr(IV) concentration ions versus time was given for three different phases as a donor, acceptor, and organic phase. The graph of the extraction kinetics of Zr(IV) ions for carrier concentration is shown in Figure 3.

Extraction of 100 mg L^−1^ Zr(IV) from 0.50 M H_2_SO_4_ solution in the donor phase was performed with varying amounts of TOA from 0.05 mL to 0.50 mL dissolved in kerosene. The graph of the yield versus time of *C*_e_ mg L^−1^ demonstrates the incorporation of four amine extractant molecules into the extracted zirconium complex (Figure 2). Zirconium was extracted by all amines at high sulfuric acid concentration reported by Schrotterova et al. [11].

For the concentration of 0.05, 0.10, 0.25, and 0.50 mL TOA, Zr(IV) transportation yields were 99.4%, 99.3%, 99.9%, and 99.8%, from donor phase to acceptor phase, respectively. The highest extraction efficiency (99.8%) of Zr(IV) ions transportation was evidently achieved by the organic phase with 0.50 mL TOA in kerosene.

The ln*C*_o_/*C*_e_ values against the time of the extraction of four different TOA and Zr(IV) ions from the donor phase into the organic phase are given in Figure 3 and the data regarding the kinetic calculations are given in Table 2.

As the amount of TOA dissolved in kerosene increases, the value of the reaction rate constants (*k*_1_) increases in extraction from the donor phase to the organic phase. It can be seen from the kinetic calculations that the extraction of Th(IV) ions from the donor phase to the organic phase and from the organic phase to the acceptor phase is a consecutive first-order reaction. The fact that the *R*^2^ values in Figure 3 are close to one shows the full compatibility of the obtained data with each other. Other calculated kinetic data show regular increase or decrease of *k*_2_, $t_{\text{B}}^{maks},C_{\text{B}}^{\text{maks}},J_{\text{a}}^{\text{mak}}$, and $J_{\text{d}}^{\text{mak}}$. It is due to the incomplete separation of Zr(IV) ions from TOA in the acceptor phase during the transition of the organic phase from the acceptor phase or the accumulation of Zr(IV) ions in the organic phase.

As the amount of TOA dissolved in kerosene increases, the value of the reaction rate constant (*k*_1_) which is the extraction rate constant from the donor phase to the organic phase rises, too. Accordingly, TOA concentration was chosen as 0.10 mL to study the following parameter and 99% of each TOA effluent was recovered for economical and environmental purposes.

### 3.2. Effect of temperature on the transport of Zr(IV)

**Figure 4:**
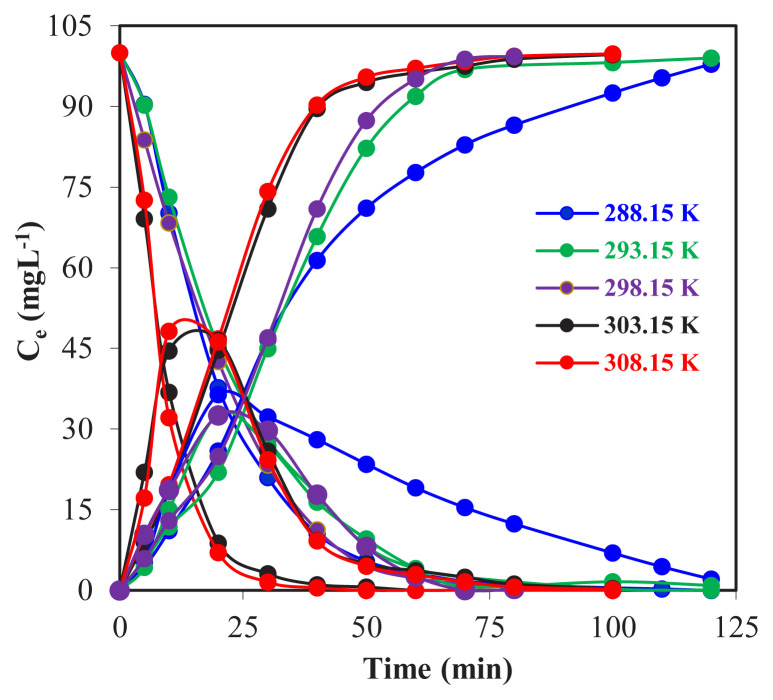
Concentrations of Zr(IV) ions in the phases versus time for the experiments carried out at different acceptor phase concentrations.

In the investigation of the effect of temperature on the extraction of Zr(IV) ions with the MDLM technique; donor, acceptor, and organic phase volumes of 100 mL, the initial Zr(IV) ion concentration of donor phase 100 mg L^−1^ in 0.5 M H_2_SO_4_ solution, 0.10 M Na_2_CO_3_ concentration in acceptor phase, TOA concentrations dissolved in organic phase kerosene 0.10 mL, the solution transfer rate of the peristaltic pump was fixed to 25 mL min^−1^ and the ambient temperature was set to 288.15, 293.15, 298.15, 303.15 and 308.15 K for each trial. The graphs of the concentrations of Zr(IV) ions versus time are given in Figure 4.

**Figure 5:**
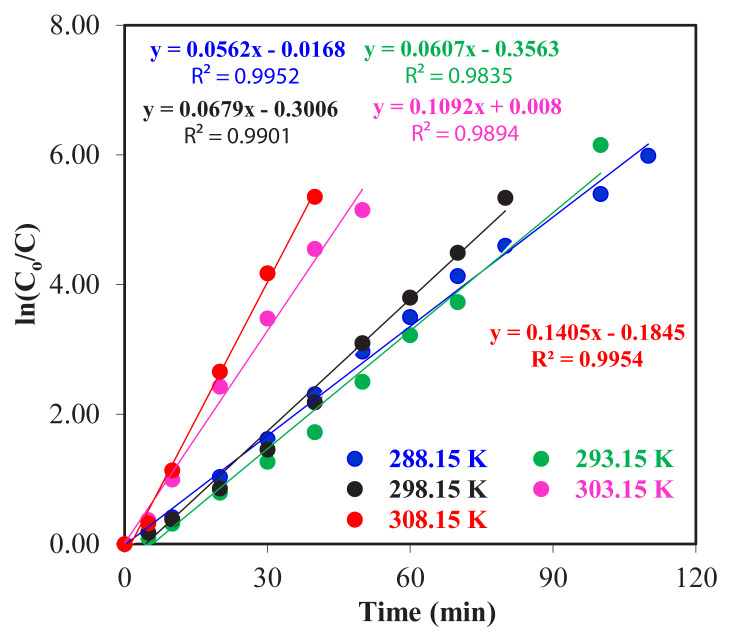
Graph of time versus ln(*C*_o_/*C*_e_) for five different temperatures.

From Figure 4, the reuptake efficiency of Zr(IV) ions from the donor phase to the organic phase and from the organic phase to the acceptor phase for different temperatures (288.15 293.15, 298.15, 303.15, and 308.15 K) was found to be 97.9%, 99.0%, 99.3%, 99.7%, and 99.8%, respectively. The transfer of Zr(IV) ions to the acceptor phase took a long time at low temperatures and shorter at high temperatures. During the interphases transportation of the ligand carrier, TOA, and Zr(IV) ions droplets, the change in temperature may alter the viscosity of the liquid mixture and this might cause changes in reupdating time and yields. In Figure 5, the graph of ln (*C*_o_/*C*_e_) versus time for five different temperatures and the kinetic data calculated for Zr(IV) ions are given in Table 2.

**Figure 6:**
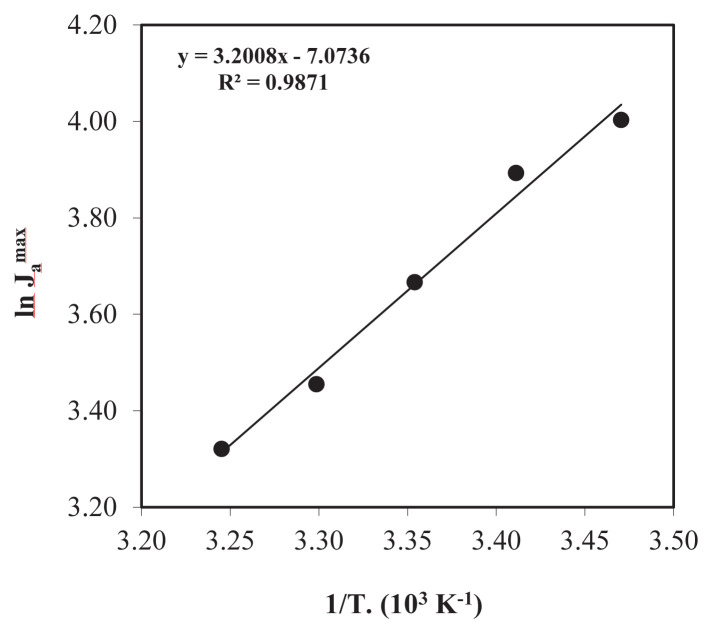
1/*T* versus ln *J*_a_^mak^ graph for five different temperatures (288.15–308.15 K).

According to the data obtained in Figure 6, *k*_1_ for different temperatures (T: 288.15, 293.15, 298.15, 303.15, and 308.15 K) was calculated as 5.62 × 10^−2^, 6.04 × 10^−2^, 6.80 × 10^−2^, 10.92 × 10^−2^ and 14.05 × 10^−2^ min^−1^, respectively. It has been proven that as the temperature increases, the reaction rate constant, *k*_1_, also increases, and *R^2^* values for each temperature are >0.98 means that the obtained data are compatible.

In order to calculate the kinetic data, transportation is supposed to occur at an average reaction rate. Experimental results suggest that the transport of Zr(IV) ions could occur at an average rate and the optimum study temperature is determined to be 298.15 K for extraction of Zr(IV) ions.

Examining the membrane entry and exit times, $J_{a}^{mak}$ *and* $J_{d}^{mak}$ of Zr(IV) ions at five different temperatures in Table 2 given below shows that they increase by the temperature elevation and $t_{B}^{maks}$ values are decreased accordingly.

The graph of 1/*T* versus ln $J_{a}^{mak}$ for five different temperatures is given in Figure 6. Equation (2.10) provided the calculation of the activation energy *(E_a_)* value for the extraction of Zr(IV) ions and the maximum membrane exit rates $J_{a}^{mak}$ against the 1/*T* values were plotted for different temperatures, and the activation energy was found by the slope of the graph as 6.36 kcal mol^−1^. Less than 10.0 kcal mol^−1^ activation energy indicates the transport mechanism of Zr(IV) ions in the diffusion-controlled system [17]. Since the only factor affecting average kinetic energy, the temperature is an important parameter in the extraction of Zr(IV) ions by the MDLM technique and the optimum temperature was chosen as 298.15 K for the system.

### 3.3. The effect of Na2CO3 concentration in the acceptor phase

Back-extraction of the acceptor phase by stripping the metal ion takes place from the metal-loaded organic phase and the organic phase allows reuse in further experiments. The extraction process becomes commercially important if the metal can be withdrawn from the charged organic phase [18].

**Figure 7:**
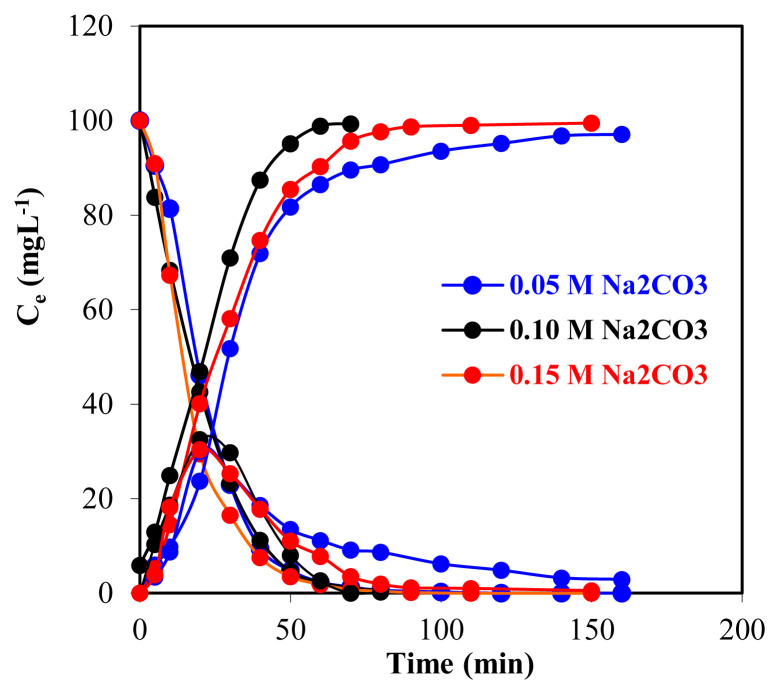
Na_2_CO_3_ concentration in three different acceptor phases and Zr(IV) ions concentration in three phases over time.

The experiment was conducted for 0.05, 0.10, and 0.15 M Na_2_CO_3_ in the acceptor phase by keeping the other parameters (pH of donor phase, volume of all three phases, temperature, TOA concentration, transfer rate of organic phase) constant and the time course data of the concentrations of Zr(IV) ions in the three phases for different Na_2_CO_3_ concentrations of the acceptor phase are given in Table 2 and the graph is given in Figure 7.

**Figure 8:**
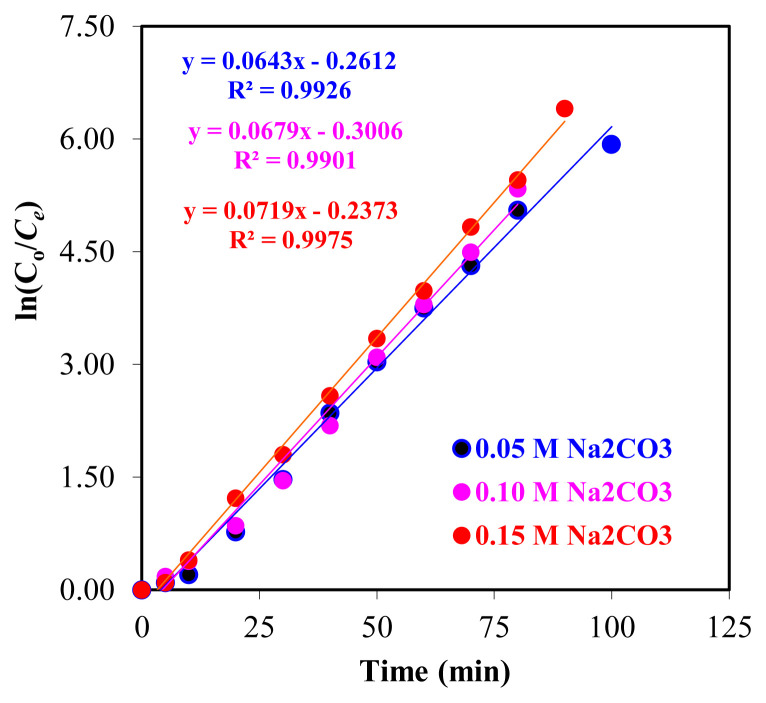
The graph of the extraction kinetics of Zr(IV) ions for experiments performed at different Na_2_CO_3_ concentrations (0.05–0.15 M).

When the stripping time was increased from the 1st min to the 60th min, the extraction percentage increased to >85% and it can be seen from Figure 8 that it remained stable after the 8th min and reached equilibrium. The uptake of Zr(IV) ions into the acceptor phase takes longer at the concentration of Na_2_CO_3_ solution other than 0.10 M.

According to the data obtained in Figure 8, the *k*_1_ values (for 0.05, 0.10, and 0.15 M Na_2_CO_3_) of the extraction of Zr(IV) ions in the donor phase were found to be 6.43 × 10^−2^, 6.79 × 10^−2^, and 7.33 × 10^−2^ min^−1^, respectively. The fact that as the concentration of Na_2_CO_3_ in the acceptor phase increases, the reaction rate constant, *k*_1_ values also increase, and R^2^ > 0.99 values for each concentration indicate that the obtained data are compatible with each other.

It has been determined that Zr(IV) ions can be transported even at low Na_2_CO_3_ concentrations via the MDLM system. Quantitative backextraction of both zirconium and hafnium from loading TOA in a low concentration Na_2_CO_3_ solution was reported by Wang and Lee [19].

The Zr-depleted organic phase can be removed by the following reaction with Na_2_CO_3_ solution in the organic regeneration stages [20]. The probable stripping reaction with Na_2_CO_3_ may be represented as


(12) $${\left({\text{R}}_{3}\text{NH}\right)}_{2}\text{Zr}{\left({\text{SO}}_{4}\right)}_{3}+2{{\text{Na}}^{+}}_{\left(\text{aq}\right)}+{{\text{CO}}_{3}^{-}}_{\left(\text{aq}\right)}\to 2{\text{R}}_{3}{\text{N}}_{\left(\text{org}\right)}+4{{\text{Na}}^{+}}_{\left(\text{aq}\right)}+{{\text{Zr}}^{4+}}_{\left(\text{aq}\right)}+2{{\text{SO}}_{4}^{2-}}_{\left(\text{aq}\right)}+{\text{H}}_{2}{\text{O}}_{\left(\text{aq}\right)}+{\text{CO}}_{2(\text{g})}.$$

### 3.4. The effect of H2SO4 concentration in the donor phase

Since H_2_SO_4_ concentration determines the acidity of the donor phase, it is one of the most significant factors for the extraction of Zr(IV) ions. The extraction behavior of Zr(IV) ions was investigated through different concentrations (0.50, 0.75, 1.00, and 1.25 M) of the H_2_SO_4_ solution of the donor phase at 298.15 K. The extraction was performed by the use of the concentration of acceptor phase as 0.10 M Na_2_CO_3_, the concentration of TOA as 0.50 mL, the volume of feed, organic and stripping solutions as 100 mL.

**Figure 9:**
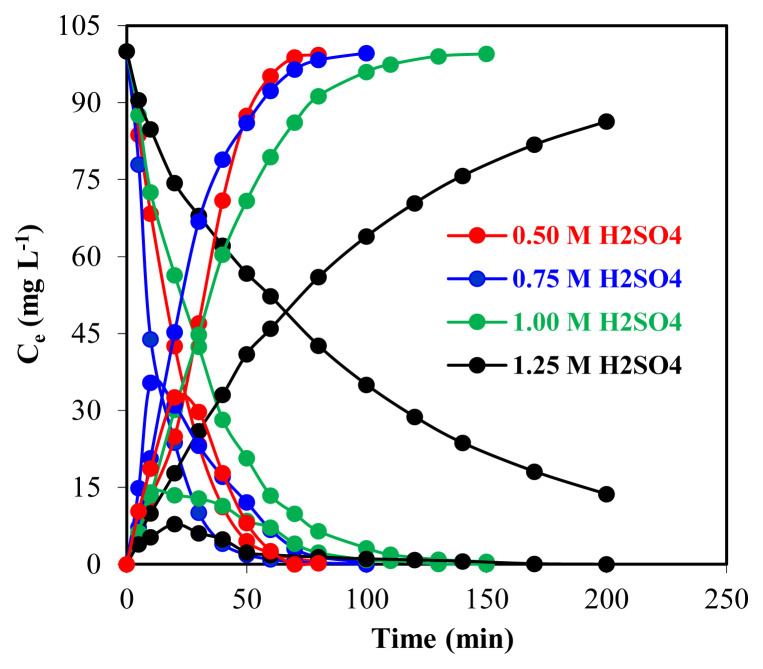
H_2_SO_4_ concentration in four different donor phases and Zr(IV) ions concentration in four phases over time.

In the experiments carried out by different initial acceptor phases (0.50–1.25 M H_2_SO_4_), transport yields of Zr(IV) ions from the donor phase to the acceptor phase were found as 99.3%, 99.7%, 99.5%, and 86.3%, respectively. Zr(IV) concentration of feed, membrane and acceptor phase solutions against the time plot are reported in Figure 9. The decrease in extraction with increasing acidity could be due to the sulfate and bisulfate competition.

The extraction of Zr(IV) was studied with 0.10 M TOA in kerosene by varying H_2_SO_4_ concentration from 0.50 to 1.25 M. Extraction was 99.7% with 0.75 M H_2_SO_4_ and then decreased up to 86.3% with 1.25 M H_2_SO_4_. In the low acid concentration (0.50 to 1.00 M), problems such as turbidity did not occur, and phase separation was easy. The extraction was observed to depend on the acidity of the aqueous phase due to the formation of more amine salts that extract the metal complex. It was determined that as the concentration of H_2_SO_4_ in the donor phase increased, the extraction efficiency and the extraction time increased, too.

It seems that the possible extraction mechanism of Zr(IV) from H_2_SO_4_ environment with TOA in kerosene proceeds via protonation of TOA (R_3_N) to form R_3_NHSO_4_ followed by extraction of (R_3_NH) ZrOCl_2_ species into the organic phase. From its results under the studied experimental conditions, the extraction of Zr(IV) from the acidic chloride medium by TOA can be generally described by the following Eq. (13) [21].


(13) $${\text{ZrOCl}}_{2}(\text{aq})+{\text{R}}_{3}{\text{NHSO}}_{4}\;(\text{org})⇌{\text{R}}_{3}{\text{NHZrOSO}}_{4}(\text{org})$$

**Figure 10:**
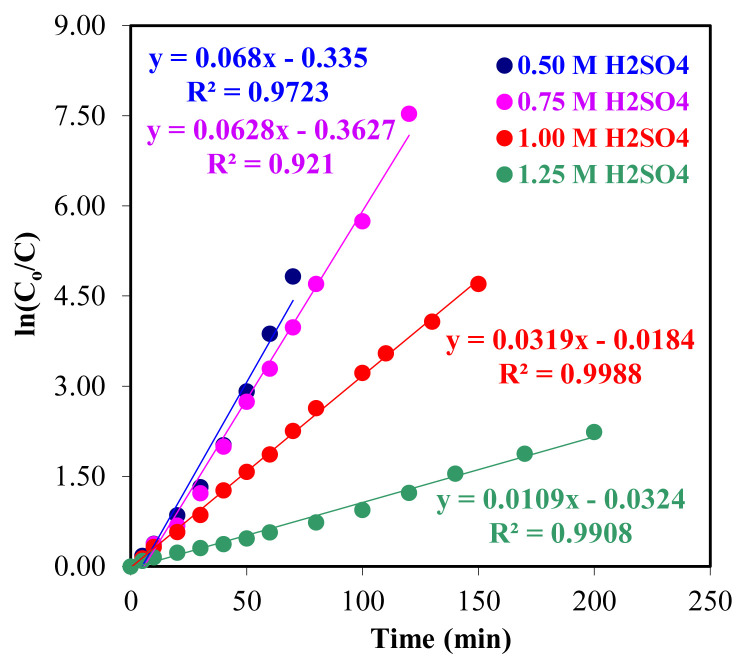
1/*T* versus ln *C*_o_/*C*_e_ plot for four different H_2_SO_4_ concentrations.

The extraction and stripping of the Zr(IV) ions treatments conform to the successive first-order reaction kinetics according to the change of ln (*C*_o_/*C*_e_) with the time plots in Figure 10. In four different concentrations of H_2_SO_4_ solution (0.50–1.25 M) in donor phase, *k*_1_ is 6.79 *×* 10^−2^, 7.46 *×* 10^−2^, 3.63 × 10^−2^ and 1.00 × 10^−2^ min^−1^, respectively. Other kinetic data calculated for these parameters of zirconium ions are given in Table 2.

### 3.5. Selective transportation of Zr(IV) ions

Contemporaneous batch test has been carried out by using 50 mg L^−1^: Zn(II), Cu(II), Cd(II), Co(II) and Ni(II) ions by MDLM system at optimum conditions as 0.10 M Na_2_CO_3_ concentration in acceptor phase, 0.50 M H_2_SO_4_ concentration of donor phase, 0.10 mL TOA concentration of 100 mL organic phases, pH: 0.50, 25 mL min^−1^ rates of peristaltic pump, at 298.15 K.

The analyzes of each metal ion with Zr(IV) were measured with an ICP device in Pamukkale University ILTAM laboratory. It was observed that other metal ions other than Zr(IV) ions and cadmium ions did not pass into the acceptor phase in the acceptor phase. In the presence of other metal ions, Zr(IV) ions were extracted to >99% acceptor phase under optimum conditions, while 0.424 mg L^−1^ (0.85%) Cd(II) ions were also extracted. Besides zirconium ions, Zn(II), Cu(II), Co(II), and Ni(II) can be extracted selectively, but the extraction of cadmium ions into the acceptor phase shows that the study is not selective.

Binary metal ion extraction was investigated by placing 100 mg L^−1^ Zr(IV) and 100 mg L^−1^ Cd(II) in the donor phase in the MDLM system under optimum conditions. Only 0.433 mgL^−1^ Cd(II) ions and 99.10 mg/L^−1^ Zr(IV) ions were extracted into the acceptor phase. Similar studies were carried out separately for Zn(II), Cu(II), Co(II), and Ni(II) ions. These metal ions along with Zr(IV) ions were not extracted into the acceptor phase. It has been observed that Zr(IV) ions can be selectively extracted in the presence of Zn(II), Cu(II), Co(II), and Ni(II) ions under optimal conditions at the same concentrations with the MDLM system.

From the study on the extraction of zirconium from the donor phase with the MDLM method selected using extractor TOA from the organic phase and internal acceptor phase containing a stripping agent so that the extraction and reextraction run simultaneously. When compared with the usual extraction method, namely liquid-liquid extraction, there are savings in terms of time and cost. It is clear that Zr(IV) ions can be extracted selectively in the presence of other metal ions with a high yield.

Comparison of some reuptake efficiencies of the extraction of single and selective zirconium ions with other techniques is given in Table 3.

Table 3 shows that the extraction efficiency of Zr(IV) ions is the highest with the MDLM system compared to other extraction systems.

The extraction conditions and recovery efficiencies of other heavy metals by the MDLM technique are given in Table 4.

Table 2 shows that some heavy metal ions with different valences have high extraction efficiencies with the MDLM system under optimal conditions. It is clear that the extraction of heavy metal ions from an aqueous solution with different carrier ligands using the MDLM system can be effective with this system.

**Figure 11:**
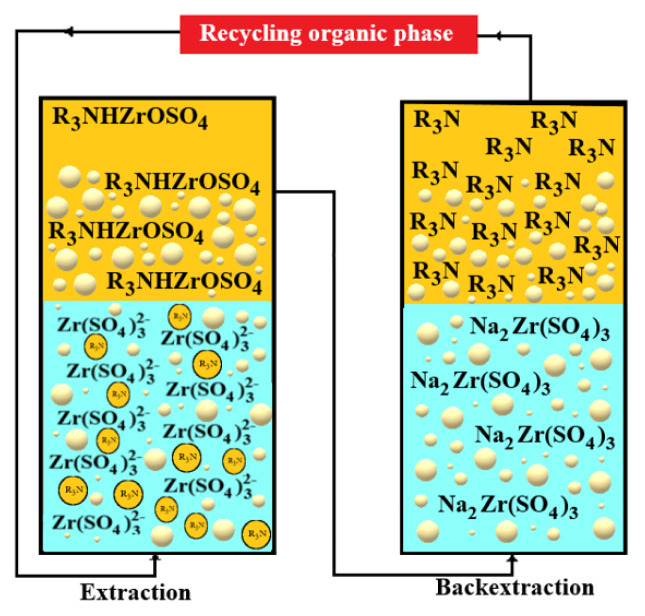
The reaction mechanism of Zr(IV) with carrier ligands in donor, organic, and acceptor phases in MDLM system.

The chemical mechanism of the transport of Zr(IV) ions by TOA in the MDLM system used in our study is given in Figure 11.

## 4. Conclusion

Factors (TOA solution concentration, temperature, H_2_SO_4_ in donor phase and Na_2_CO_3_ concentration effect used as acceptor phase) affecting the reaction mechanism of extraction were investigated by using TOA solution dissolved in kerosene to transport Zr(IV) ions from the donor phase to the acceptor phase using the MDLM system.

- The optimum conditions were determined experimentally and Zr(IV) was extracted with an extraction efficiency of about >99.5%.
- TOA dissolved in kerosene was used as the carrier ligand.
- Reactor type having a glass filter with porosity number 0.0 was used. The larger the porosity node of the glass filter, the smaller the pore size. Thus, the size of the membrane droplets formed in the acceptor and donor phases decreases, the total membrane surface area in contact with the aqueous phase increases, and the flow rate increases partially due to pressure. However, best Zr(IV) ions transportation was managed by using glass filters with porosity number 0.0.
- The change and reuptake yield of Zr(IV) ions in donor, acceptor, and organic phases over time were calculated. By the MDLM technique, the activation energy for five different temperatures was calculated as 6.36 kcal mol^−1^, and it was determined that the transport mechanism for the extraction of Zr(IV) ions was diffusion-controlled.
- These investigations propose that the TOA can be successfully used as a carrier for the removal of toxic Zr(IV) ions from inappropriate wastewater treatment systems.
- It has been determined that the extraction of Zr(IV) ions, performed under optimum conditions, in the presence of other metal ions [Zn (II), Cu (II), Cd (II), Co (II) and Ni (II)] was found to be selective. Extraction of Th(IV) ions is not selective in the presence of Cd metal ions under optimal conditions.

To summarize the results briefly, efficient outcomes can be obtained using the carrier ligands and the experimental setup of the liquid membrane system.

## Figures and Tables

**Table 1:** Extractors with some amine group content used in the extraction of Zr(IV) ions.

Extractant	Reference
**Primary amines**	
Primene JMT	[11,12]
**Secondary amines**	
Di-cyclohexyl amine	[13]
Di-n-octyl amine	[14]
Di-n-nonyl amine	[11,12]
**Tertiary amines**	
Tri-n-octyl amine (Alamine 336)	[14]
Tri-n-dodecyl amine	[14]
Tris-(2-ethylhexyl) amine	[14]
**Quaternary ammonium salts**	
Tri-caprylmethylammonium chloride	[11]

**Table 2:** Calculated kinetic data for the extraction of Zr(IV) ions with the MDLM system under different conditions.

		*k*_1_.10^2^ (min^−1^)	*k*_2_.10^2^ (min^−1^)	(min)	(mgL^−1^)	10^2^ (min)	10^2^ (min)
**TOA (mL)**	0.05	6.59	14.84	9.84	23.22	3.45	−3.45
	0.10	6.79	6.77	14.75	36.84	2.49	−2.49
	0.25	9.00	38.97	4.89	14.87	5.80	−5.80
	0.50	10.16	8.23	10.92	40.73	3.35	−3.35
**Temperature (K)**	288.15	5.62	4.42	19.62	42.02	1.82	−1.82
	293.15	6.04	5.10	17.99	39.93	2.04	−2.04
	298.15	6.80	7.10	14.39	36.00	2.55	−2.55
	303.15	10.92	6.98	11.36	45.26	3.16	−3.16
	308.15	14.05	7.35	9.67	49.13	3.61	−3.61
**Na** **_2_** **CO** **_3_** ** (M)**	0.05	6.43	7.50	14.39	34.00	2.55	−2.55
	0.10	6.79	7.10	14.40	35.97	2.55	−2.55
	0.15	7.33	6.10	14.93	40.21	2.45	−2.45
**H** **_2_** **SO** **_4_** ** (M)**	0.50	6.79	7.10	14.40	35.97	2.55	−2.55
	0.75	7.46	7.13	13.55	38.07	2.71	−2.71
	1.00	3.63	11.12	14.89	18.89	2.11	−2.11
	1.25	1.00	9.58	26.34	8.02	0.77	−0.77

**Table 3:** Extractant used in Zr(IV) metals extraction using conventional solvent extraction (SE), bulk liquid membrane (BLM), liquid emulsion membrane (LEM), liquid-liquid extraction (LLE) emulsion liquid membrane (ELM), liquid surfactant membrane (LSM), supported liquid membrane (SLM) processes.

System	Internal liquor	Extractant	Diluent	Surfactant	Removal (%)	References
BLM	HCl	TBP	Kerosene	-	97.7	[22]
SE	H_2_SO_4_	Alamine 308	-	-	90	[23]
LLE	H_2_SO_4_	TOA	Kerosene	-	97.4	[24]
LEM	HNO_3_	Cyanex-272	Corn oil	Span 80	91.8	[25]
SE	HC/KF	2-Octanol		-	93.1	[26]
SLM	HCl	TNOA/Aliquat 336	-	-	85	[27]
SE	HNO_3_	Cyanex 925	-	-	91.6	[28]
LLE	HCl	Alamine 308	-	-	97.8	[29]
SE	HNO_3_	TBP	-	-	67	[30]
SE	Mix Acid	D2EHPA	-	-	71	[31]
SE	HNO_3_	LIX 63	-	-	92	[32]
SE	Oxalic acid	Cyanex 923	-	-	98	[33]
SE	HCl	PC-88A	-		85.7	[3]
SE	HNO_3_	PC 88A and Lix 63	-	-	93	[35]
SE	HCl	LIX 63 and Cyanex 272	-	-	88.6	[36]
MDLM	H_2_SO_4_	D2EHPA	Kerosene	-	99.5	This Study

**Table 4:** Reuptake efficiencies data in the extraction of other metal ions with the MDLM. system.

System	Donor phase	Extractant/diluent	Acceptor phase	Metal ions	Removal, %	References
MDLM	HCl	D2EHPA/kerosene	HCl	Zn(II)	99.9	[1]
MDLM	Na_2_B_4_O_7_ and NaOH	TNOA/kerosene	Na2CO_3_	Mo(VI)	90.1	[8]
MDLM	CH_3_COOH and CH_3_COONa	D2EHPA/kerosene	H_2_SO_4_	Cu(II)	99.8	[9]
MDLM	NH_3_	TNOA/kerosene	H_2_SO_4_	Cu(II)	99.9	[37]
MDLM	HNO_3_	TOPO/kerosene	H_2_SO_4_	Th(IV)	98	[38]
MDLM	HCl/NaCl	TNOA/kerosene	CH_3_COONH_4_	Cd(II)	99.5	[39]
MDLM	HCl/NaOH	D2EHPA/kerosene	HNO_3_	Pb(II)	98.8	[40]
MDLM	HNO_3_	D2EHPA/kerosene	NH_4_HCO_3_	U(VI)	96.5	[41]
